# Investigations on Stability of Polycarboxylate Superplasticizers in Alkaline Activators for Geopolymer Binders

**DOI:** 10.3390/ma16155369

**Published:** 2023-07-31

**Authors:** Stephan Partschefeld, Adrian Tutal, Thomas Halmanseder, Jens Schneider, Andrea Osburg

**Affiliations:** Chair of Construction Chemistry and Polymer Materials, F.A. Finger Institute of Building Materials Science, Bauhaus-Universität Weimar, 99423 Weimar, Germany; adrian.tutal@uni-weimar.de (A.T.); thomas.halmanseder@uni-weimar.de (T.H.); jens.schneider@uni-weimar.de (J.S.); andrea.osburg@uni-weimar.de (A.O.)

**Keywords:** superplasticizer, polycarboxylate ether, metakaoline, alkaline activators, rheology, alkali hydroxide solutions, alkali silicate solutions, degradation process

## Abstract

Calcined clays are interesting starting materials to be used as SCMs (supplementary cementitious materials) in cements or to be converted to geopolymers by activation with a high alkaline activator. The adjustment of the properties in the fresh state, especially regarding the consistency of these binders, is almost exclusively achieved by the addition of water, since commercially available superplasticizers seem to be ineffective in low-calcium geopolymer systems. The aim of this study was a systematic investigation of various PCE (polycarboxylate ester/ether) superplasticizers (methacrylate ester PCE: MPEG, isoprenol ether PCE: IPEG, methallyl ether PCE: HPEG) with respect to their stability in different alkaline activators (NaOH, KOH, sodium and potassium silicate solutions). The effectiveness of superplasticizers (SPs) in low-calcium geopolymer binders was verified by rheological tests. Size exclusion chromatography was used to investigate if structural degradation of the superplasticizers occurs. The investigated PCE superplasticizers showed a thickening effect in the low-calcium geopolymer system. Depending on the alkalinity of the activator solution, a degradation process was detected for all the PCEs investigated. The side chains of the PCEs are cleaved off the backbone by basic ester and ether hydrolysis. The highest degree of degradation was found in sodium and potassium silicate solutions. In alkaline hydroxide solutions, the degradation process increases with increasing alkalinity.

## 1. Introduction

New binder systems for the building materials industry are needed to reduce the high CO_2_ emissions from cement production, which are responsible for over 8% of anthropogenic CO_2_ emissions [1,2]. A promising way for more environmentally friendly cement is the use of secondary cementitious materials, which include fly ash, ground granulated blast furnace slag (GGBS), silica fume, natural pozzolana and natural calcined pozzolana. In particular, calcined clays can be dehydroxylated at low temperatures (550–800 °C) and used as supplementary cementitious materials (SCMs) to reduce the amount of Portland cement clinker [3,4,5]. The suitability depends on their pozzolanic reactivity and their global availability. However, the substitution of cement for calcined clays often leads to reduced workability of concrete [6,7]. By using alkaline activators like NaOH, KOH or alkaline silicate solutions, the calcined clays form alkali-activated binders (AABs) or geopolymers [8]. In particular, low-calcium geopolymer binders are characterized by high stability to acid attack, high strength and durability [9,10,11,12]. A major problem in the use of calcined clays and other SCMs is their high-water demand, resulting from a high fineness and specific surface [13], which increases the viscosity and yield strength of the binders. Usually, superplasticizers (SPs), like polycarboxylate ethers (PCEs) or polycondensates (PCs), are used to control and adjust the rheological properties of cementitious materials in the fresh state [14,15]. When superplasticizers are used in SCM blended cement, there is an adsorption competition between the SCMs and the cement for the superplasticizer [16,17]. In particular, the use of calcined clays as SCMs can limit the dispersing performance of PCE-based superplasticizers by intercalation of the side chains [18,19]. For this reason, effective superplasticizers are of enormous importance in these binder systems to adjust the processing properties in the fresh state [20,21,22,23]. Therefore, they represent a key technology that enables the production of durable concrete made with SCMs or geopolymers. Kashani et al. conducted studies on alkali-activated slag paste with superplasticizers of different architectures. A high side chain density seems to be beneficial for dispersion performance [24]. Sposito et al. investigated the effectiveness of polycondensate and PCE-based superplasticizers on different blended cementitious systems to evaluate the demand for superplasticizers depending on the type of cement replacement [25]. They found that the mineralogical composition of calcined clays is decisive for the demand for superplasticizers. Furthermore, low-calcium geopolymers showed incompatibility or reduced efficiency with water-reducing admixtures, like superplasticizers [26,27]. The adjustment of the properties in the fresh state, especially regarding the consistency of these binders, is almost exclusively achieved by the addition of water [28]. Favier et al. investigated the flow properties of metakaolin-based geopolymers. The rheological parameters of pastes are controlled only by the viscosity of the alkaline solutions [29]. Although many investigations of SPs have been carried out in Portland cement, only a few studies have been carried out in AABs with various activators [23,30,31,32,33,34]. Studies on NaOH-activated slags have shown that competitive adsorption occurs between the negatively charged activator and the anionic anchor groups of the superplasticizer. This leads to reduced adsorption of the superplasticizers on the surface of slags, which reduces the efficiency of the superplasticizer [14,15]. Investigations by Luukkonen et al. on alkali-activated slags showed that lignin sulfonate- and polycondensate-based plasticizers are more efficient than PCEs [35]. Palacois et al. investigated the influence of naphthalene-, melamine- and vinyl copolymer-based superplasticizers in NaOH-activated slags. The authors found that only naphthalene-based superplasticizers reduced the yield stress and justified this to higher chemical stability [36]. Furthermore, they found that, in particular, in alkali-activated slag suspensions that were activated by sodium silicates, all the superplasticizers were ineffective. Another reason for the low effectiveness of existing superplasticizers (sulphonated melamine formaldehyde, SMF and sulphonated naphthalene formaldehyde, SNF) is their stability to alkaline activators. Studies have shown that SMFs are degraded by up to 65% at pH = 14. SNF shows higher stability and was degraded by up to 10% [37]. PCE superplasticizers also show insufficient effectiveness in AAB systems [17,18,19]. Schmid and Plank investigated the dispersing performance of calcined clay blended cement. They found that calcined clays increased the water demand and that the PCE efficiency decreased markedly at a substitution rate of 50% [38]. Methallyl ether-based PCEs were found to be more effective. In particular, the amide and ester groups, linking the side chains to the backbone of the superplasticizers, tend to be hydrolyzed in a high-pH medium [33,39,40]. Investigations of superplasticizers in geopolymer pastes based on metakaolin and sodium silicate solutions showed that, in particular, PCE does not work [41]. The aim of this study was a systematic investigation of the dispersing functionality of PCEs in low-calcium geopolymers. The work will focus on the stability of ester and ether-type polycarboxylate superplasticizers (MPEG, HPEG and IPEG PCEs) in alkaline activators. In particular, the type (NaOH, KOH sodium and potassium silicate solutions), alkalinity and exposure time in the alkaline activator, which are typically used to form low-calcium geopolymer binders, have been varied.

## 2. Materials and Methods

### 2.1. Materials

**Figure 1:**
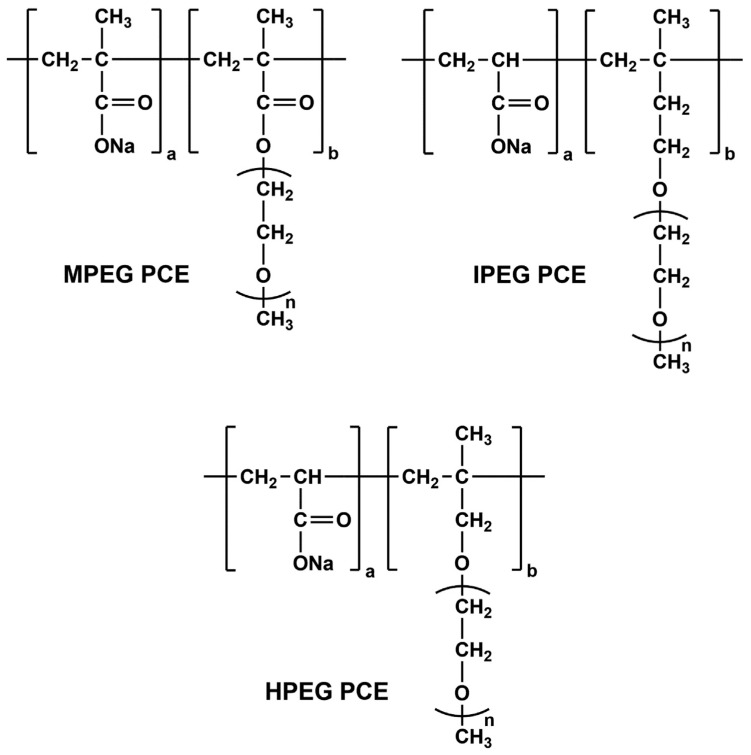
Chemical structure of HPEG-, IPEG- and MPEG-type PCEs [38].

The investigated PCE superplasticizers were provided by MBCC Group (Master Builders Solutions Deutschland GmbH, Mannheim, Germany) and varied by their chemical architecture (Figure 1) in the form of HPEG-, IPEG- and MPEG-type.

**Figure 2:**
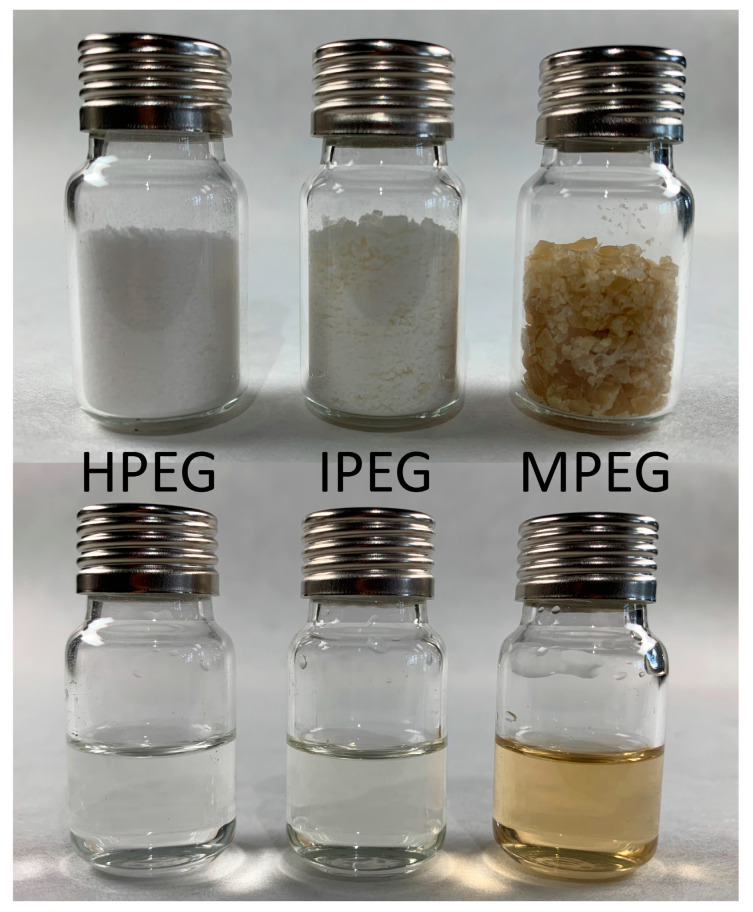
Dry samples (**top**) and 10 wt.-% solutions (**bottom**) of investigated PCEs.

The PCEs were supplied in liquid form. To characterize the molecular weight, the three PCEs were lyophilized until mass consistency was obtained and ground with a mortar and pestle. While HPEG- and IPEG-PCEs lead to fine white powders, the drying and grinding of MPEG-PCEs lead to small yellow-brown flakes, as seen in Figure 2.

NaOH pellets (≥98%, Carl Roth GmbH + Co. KG, Karlsruhe, Germany) or KOH pellets (≥85%, Carl Roth GmbH + Co. KG) were dissolved in ultrapure water (ASTM type 1) to obtain sodium hydroxide and potassium hydroxide solutions with concentrations of 1 mol/L (1 M), 4 mol/L (4 M) and 8 mol/L (8 M). Silica gel (≥99.4%, 400–220 mesh, Carl Roth GmbH + Co. KG) was dissolved in sodium hydroxide solution (7.8 M) or potassium hydroxide solution (6.6 M) to obtain alkali silicate solutions with a solid content of 40 wt.-% and a SiO_2_/M_2_O ratio of 2. Hydrochloric acid (37 wt.-%, Carl Roth GmbH + Co. KG) was diluted with ultrapure water to different concentrations for pH neutralization of the alkaline solutions described above. For an investigation of the influence of the PCEs on the rheological properties of low-calcium geopolymer pastes, a metakaolin (Metaver O, Newchem GmbH, Baden, Austria) was used for the preparation of the pastes. The metakaolin is almost free of CaO and is mainly composed of 52.0% SiO_2_, 41.4% Al_2_O_3_, 0.9% TiO_2_, 0.6% and 0.3% K_2_O, which was measured by ICP-OES. XRD and Rietveld phase analysis showed 72% amorphous content, 24% of kaolinite, 0.9% of anatase and 2.5% of quartz.

The particle size distribution of the metakaolin was determined using laser granulometry and showed an average particle size of 8.19 µm with a d_10_ percentile of 0.75 µm and a d_90_ percentile of 20.22 µm. The specific surface area of the metakaolin was determined as ≈11.5 m^2^/g via the BET method. The modified Chapelle test described in the French norm NF P 18-513, Annexe A was used to investigate the pozzolanic reactivity of the metakaolin [42]. Since the norm requires a reaction conversion of 700 mg of Ca(OH)_2_ per gram of sample for a material to be defined as “pozzolanic”, the investigated metakaolin reactivity was 1275 ± 30 mg/g.

### 2.2. Methods

#### 2.2.1. Size Exclusion Chromatography

Size exclusion chromatography (SEC) was used to analyze the samples’ molecular weight distribution of the superplasticizers before and after stability investigations. An AF2000 MultiFlow FFF System (Postnova Analytics GmbH, Landsberg am Lech, Germany) was equipped with a Shodex OHpak SB-805 HQ SEC column (Resonac Europe GmbH, Gersthofen, Germany). The particle size of the column material was 13 µm with a pore size of 7000 Å. A refractive index (RI) detector was used for the acquisition of the sample fractions. The RI detector was calibrated using 180–708.000 Da pullulan standards in 0.05% NaN_3_ eluent (PSS Polymer Standards Service GmbH, Mainz, Germany).

A 0.05% NaN_3_ solution was used as the eluent, and the samples were prepared by the dissolution of 10 mg of sample in 10 mL of eluent. The investigations were performed at a flow rate of 0.5 mL/min. For each sample, 20 µL was injected into the SEC by an autosampler. The RI detector was used for the evaluation of the results since it was best suited to detect the polymers as well as the monomers in the sample. The accuracy of the method was investigated by measuring three samples with 1 mg/mL of PCE and three with 5 mg/mL of PCE for each type of PCE. The results of the six samples of each PCE type were then used to determine the standard deviations and coefficients of deviation for the weight and number average molar mass (M_w_ and M_n_) as well as the polydispersity index (PDI). The dn/dc value was measured by direct injection without separation by column for each PCE to determine the recovery rate. For the HPEG-PCEs, the dn/dc was determined as 0.16, and it was determined as 0.174 for the IPEG-PCEs and 0.17 for the MPEG-PCEs.

The possibility of PCEs being adsorbed by the precipitated silica gel needed to be considered for the design of the experiment. Therefore, a sample with MPEG-PCEs and a sodium silicate solution was neutralized with hydrochloric acid and centrifuged at 2215× *g* for five minutes to separate the precipitated silica gel from the supernatant. The silica gel was washed five times with ethanol (≥96%, Carl Roth GmbH + Co. KG) to wash off any adsorbed PCEs. The ethanol was evaporated, the dry residual chemicals were dissolved in 0.05% NaN_3_ solution and the sample was analyzed by SEC. Only small amounts of (macro-)monomers were found in the sample, which lead to the assumption that PCEs do not get adsorbed by the precipitated silica gel in significant amounts.

#### 2.2.2. Binder Preparation and Rheological Investigations

PCE solutions of different concentrations (0.5, 1.0 and 2.0 wt.-%) were prepared by dissolving dry PCE powders in ultrapure water. The concentration was related to the amount of binder, which is considered the amount of solid content of the alkaline activator and metakaolin. The recipe for the geopolymer pastes was designed to result in pastes with similar viscosity, regardless of whether a sodium silicate solution or potassium silicate solution was used as the alkaline activator. In total, 20 g of metakaolin was mixed with 18 g of sodium silicate solution or 16 g of potassium silicate solution, a fixed amount of water and 1 mL of PCE solution. The components were mixed with an overhead stirrer for two minutes at 400 RPM. The resulting paste’s water-to-binder ratio was 0.40 for pastes with potassium silicate solution and 0.43 for pastes with sodium silicate solution.

The rheology of geopolymer pastes was investigated by the measurement of dynamic viscosity and shear stress. A Brookfield DV-III rheometer (AMETEK GmbH, Berwyn, PA, USA) equipped with a spindle of type SC4 29 was used for the measurements. The measurement began after pre-shearing the samples at 120 RPM for 60 s. After that, the rotational speed was reduced in 9 steps to 1 RPM. Each step was fixed for 30 s, and every 5 s, the shear stress and dynamic viscosity were determined. All the examinations were performed in triplicate.

#### 2.2.3. Stability Test Preparation of PCEs in Different Alkaline Media

For chemical stability investigations, liquid samples containing 20 wt.-% of PCEs were prepared by dissolving 1 g of PCEs in 5 mL of ultrapure water. A beaker with a magnetic stirrer was filled with 99 mL of alkaline solution before 1 mL of the prepared PCE solution was added. The resulting PCE concentration in the solution was, therefore, approximately 2 mg/mL The solutions were neutralized with hydrochloric acid (HCl) after time intervals of 5, 10, 15 and 30 min, filled in round bottom flasks and lyophilized. Due to the decrease in the pH value during the addition of HCl, silica gel (SiO_2_) precipitated. Therefore, dried samples from the alkali hydroxide solution were mainly composed of sodium chloride (NaCl) or potassium chloride (KCl), while the samples from the alkali silicate solutions also contained silica gel (SiO_2_). The solubility of NaCl (0.65 g/kg) and KCl (0.37 g/kg) in ethanol is low, while SiO_2_ is insoluble in ethanol at 25 °C. Therefore, to isolate the PCEs and degradation products, 30 mL of ethanol (≥96%) was added to the dried samples, and the flasks were shaken for an hour on a horizontal shaker. The samples were transferred to tubes and centrifuged at 2215× *g* for five minutes (Eppendorf, Centrifuge 5804R, Hamburg, Germany). The samples’ supernatant was separated with a syringe and filtered into round bottom flasks through a syringe filter with a 0.45 µm mesh. The solution was diluted with 60 mL of ultrapure water and subsequently lyophilized. A 0.05% NaN_3_ solution was used as the eluent, and the samples were prepared by the dissolution of 10 mg of sample in 10 mL of eluent. The samples were then analyzed as described in Section 2.2.1.

## 3. Results and Discussion

### 3.1. PCE Characteristics

**Figure 3:**
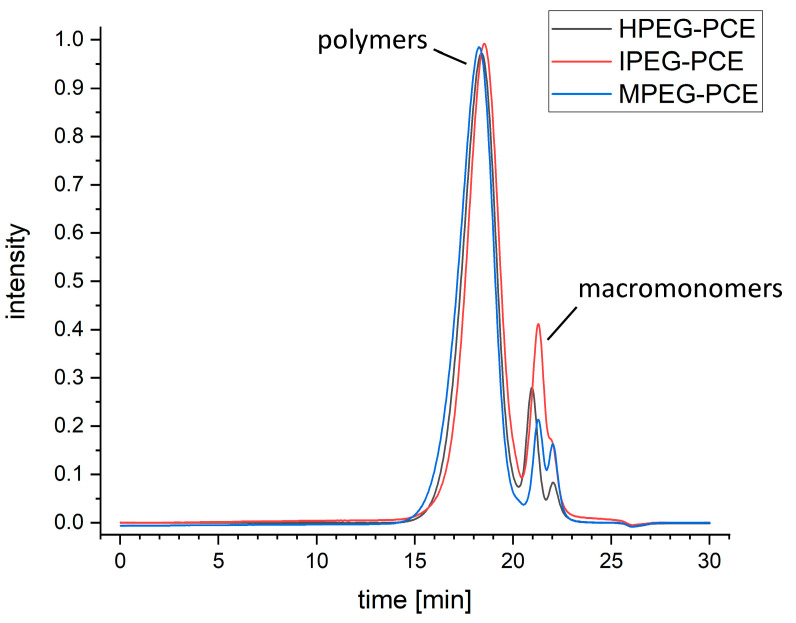
Elution curves of PCE samples.

Figure 3 shows the elution curves of all the PCE samples, while the samples’ M_w_ and M_n_, PDI and polymer content are given in Table 1. A large peak followed by a set of two smaller peaks, or in the case of IPEG-PCEs, a peak with a right-hand shoulder, can be seen for all three samples. Due to the rapid elution of polymers and macromonomers, a baseline separation is not possible with the used column. The first peak corresponds to the PCE polymers in the sample, while further peaks and shoulders can be assigned to the (macro-)monomeric residual chemicals used in the synthesis of PCEs. These residual chemicals are usually not separated from the synthesized PCE superplasticizers. The portion corresponds to methoxy polyethylene glycol (MPEG), hydroxy polyethylene glycol (HPEG) or isopropoxy polyethylene glycol (IPEG). The smaller monomer portion corresponds to polyethylene glycol, which is a byproduct of the synthesis of the macromonomer. The M_n_ and M_w_ hint at the HPEG- and MPEG-PCEs being structurally more like one another than the IPEG-PCEs, while the PDI for all the samples varied only slightly. The calculated polymer content of the samples was below 90%, meaning that over 10% of each sample is residual reactants from synthesis.

### 3.2. Impact of PCE on Rheology of Geopolymer Pastes

**Figure 4:**
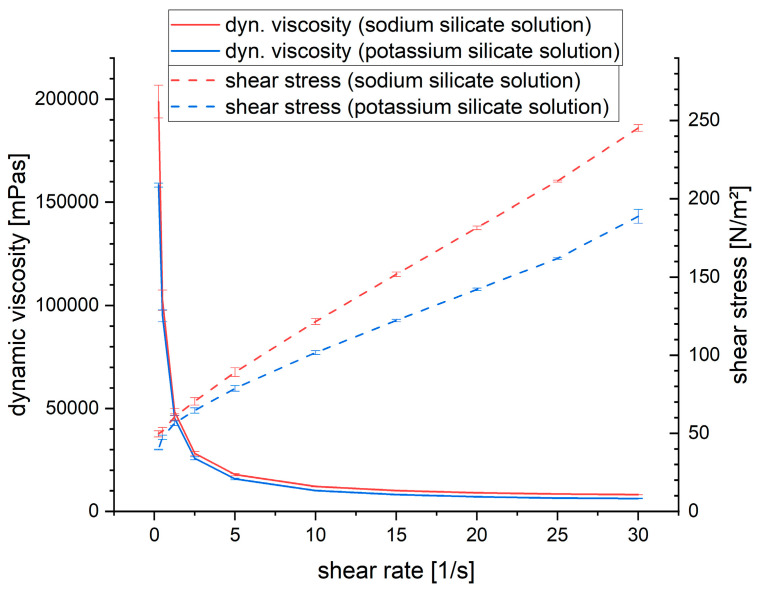
Dynamic viscosity and shear stress of reference geopolymer pastes.

**Figure 5:**
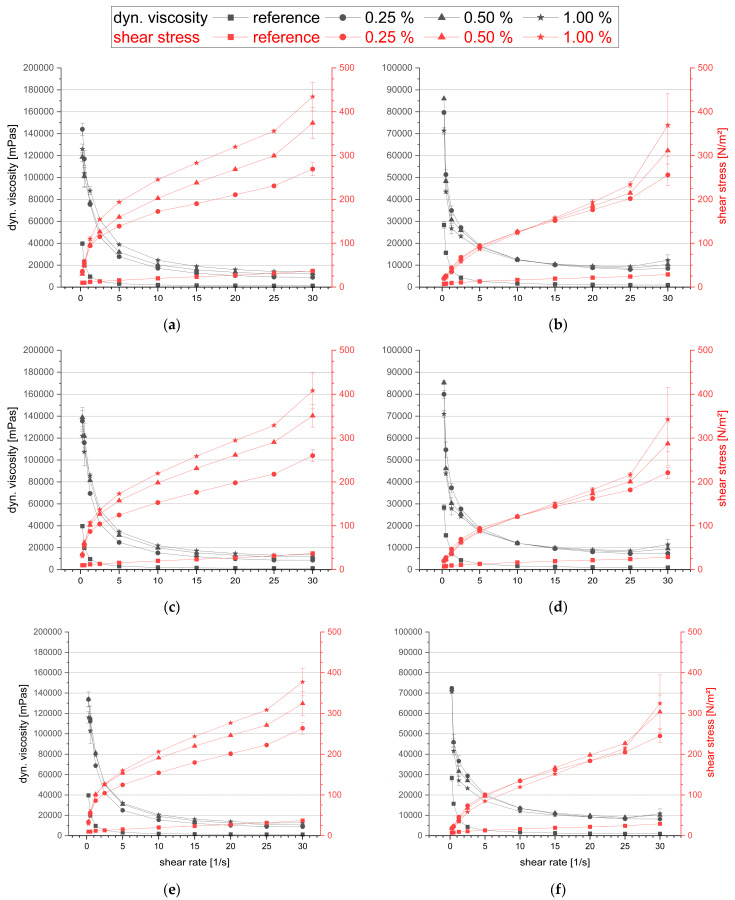
Dynamic viscosity and shear stress of geopolymer pastes made with sodium silicate solution (**a,c,e**) or potassium silicate solution (**b,d,f**) containing 0.25%, 0.5% and 1.0% of HPEG-PCEs (**a,b**), IPEG-PCEs (**c,d**) or MPEG-PCEs (**e,f**).

Two reference geopolymer pastes, one with a sodium silicate solution and one with a potassium silicate solution, were prepared. Their dynamic (dyn.) viscosity and shear stress were measured, and the average of four individual samples, together with the standard deviation, is plotted in Figure 4. The dynamic viscosity and shear stress were higher for the pastes prepared with the sodium silicate solution than with the potassium silicate solution. This is because sodium silicate solutions have a higher viscosity and shear stress by a factor of approx. 10 at comparable modulus [29,43,44]. The difference in the dynamic viscosity is high at shear rates below 1 and negligible at higher shear rates. The difference in the shear stress is only small at low shear rates and increases together with the shear rate. The dynamic viscosity and shear rates for geopolymer pastes with 0.25%, 0.5% and 1.0% are plotted in Figure 5. Note that the scale for dynamic viscosity is 0–200,000 mPa∙s for pastes made with sodium silicate solution and 0–100,000 mPa∙s for pastes made with potassium silicate solution. A significant increase in the dynamic viscosity and shear stress can be seen with the introduction of the PCEs, regardless of their type and the type of alkali silicate solution. The dynamic viscosity of all the samples containing PCEs is much higher than the reference, especially at low shear rates.

However, no clear increase in the dynamic viscosity with the PCE concentration can be seen. With an increasing shear rate, the dynamic viscosity of the samples and the difference between samples and the reference are becoming smaller. For high shear rates > 25, an increase in the dynamic viscosity can only be seen for pastes made with the potassium silicate solution. The shear stress of all the samples containing PCEs is much higher than the reference for shear rates > 1. As expected, the shear stress increases with the shear rate. However, while a clear dependency on the PCE concentration can be seen for pastes made from the sodium silicate solution for shear rates ≥ 2, the same can only be seen at high shear rates of ≥25 for pastes made with the potassium silicate solution.

The rheological studies have shown that all PCEs act as thickeners and do not cause any dispersing effect in geopolymer pastes, which is shown in Figure 5. It is assumed that PCEs are unstable in the highly alkaline environment of the geopolymer activators, and the polyethylene side chains are cleaved by hydrolysis. In addition, the free PEG side chains are thought to be responsible for the thickening effect in the geopolymer pastes. This corresponds to the results of investigations by Palacios and Puertas [45]. PCEs lose their steric repulsion forces when their PEG sidechains are cleaved off in high-alkaline media. The remaining electrostatic repulsion forces of the residual PCE backbones are superimposed by the thickening effect of the cleaved-off PEG sidechains.

To clarify this, the reference geopolymer paste was mixed with 0.1% polyethylene glycol (PEG-1000, for synthesis, Sigma Aldrich, Darmstadt, Germany). The addition of the PEG-1000 also caused the thickening of the geopolymer paste. It is assumed that the hydrolyzed PCE side chains cause the bridging of the metakaolin particles, which results in an increase in the viscosity and yield strength of the geopolymer paste.

### 3.3. Degradation in Alkali Hydroxide Solutions

**Figure 6:**
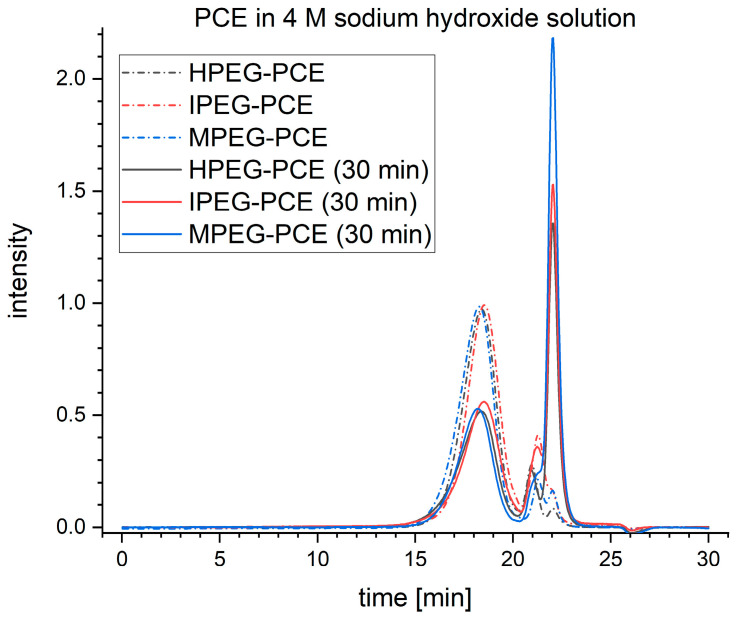
Elugrams of reference PCEs and PCE samples exposed to 4 M sodium hydroxide solution for 30 min.

**Figure 7:**
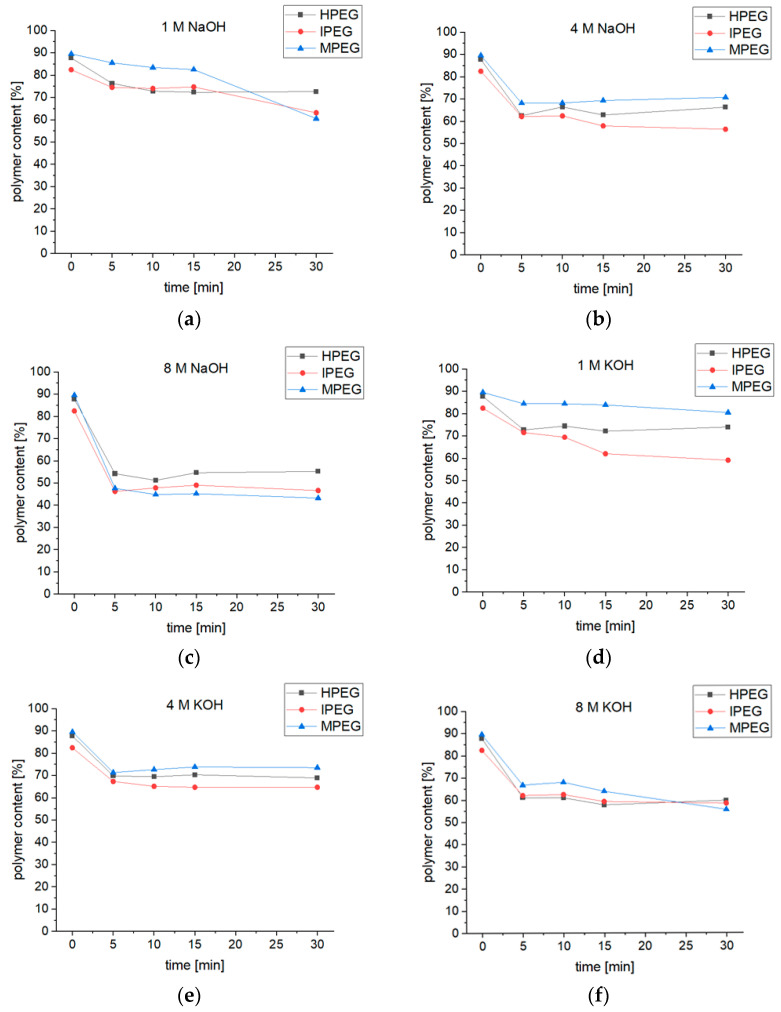
Polymer content of PCE samples depending on exposure time in (**a**) 1 M, (**b**) 4 M and (**c**) 8 M sodium hydroxide solutions and (**d**) 1 M, (**e**) 4 M and (**f**) 8 M potassium hydroxide solutions.

The elugrams for PCEs before and after 30 min of exposure to 4 M sodium hydroxide solution are given in Figure 6. All the samples show a reduction in height and width for the first peak, which corresponds to the polymeric PCE molecules after the exposure, indicating degradation of those polymers. At the same time, a much higher third peak can be seen after the exposure, which dwarfs the second peak to a mere shoulder in the case of MPEG-PCEs. Therefore, the third peak can be assigned to cleaved PEG sidechains and the second peak to macromonomers, like methoxy polyethylene glycol (MPEG), hydroxy polyethylene glycol (HPEG) or isopropoxy polyethylene glycol (IPEG). Since no further peaks are formed, the molecular weight of the PCE backbones must be like the molecular weight of the PEG sidechains. This degradation process was observed to be more dependent on the molarity of the alkali hydroxide solution rather than its type. With increasing molarity, a stronger decrease in the first peak and a stronger increase in the third peak were seen. The polymeric content of the sample was determined by assigning it to the first peak and the (macro-)monomer content to the second and third peaks of the elugram. The calculated polymer content of the samples exposed to alkali hydroxide solutions with a molarity of 1, 4 and 8 and a duration of exposure of 5, 10, 15 and 30 min are shown in Figure 7.

The degradation process of each PCE superplasticizer appears to be time-dependent in the 1 M alkali hydroxide solution. The decrease in the polymer content for the samples exposed to higher concentration solutions (4 M and 8 M) shows that most of the reaction takes place in the first five minutes of exposure, after which no significant change in the polymer content can be seen.

The degree of degradation is dependent on the molarity of the alkali hydroxide solution, with a remaining polymer content of 45–75% after 30 min of exposure. The highest degree of degradation was found in the 8 M alkali hydroxide solution. Furthermore, a difference dependent on the type of solution can be seen. The polymer content was reduced to >45% in the sodium hydroxide solution and >55% in the potassium hydroxide solution. Table 2 shows the M_n_ and M_w_ for the samples exposed to alkali hydroxide solutions for 30 min. A reduction in the M_n_ and M_w_ with increasing alkali concentration can be seen for all the samples. The values are mostly similar between the samples exposed to the sodium hydroxide solution and the potassium hydroxide solution. However, the MPEG-PCE samples’ decline in the M_n_ and M_w_ was less in the 1 M and 4 M potassium hydroxide solutions than in the corresponding sodium hydroxide solutions. As expected, the MPEG-PCEs ester bonds were less stable under alkaline conditions compared to the ether bonds of the HPEG- and IPEG-PCEs.

### 3.4. Degradation in Alkali Silicate Solution

**Figure 8:**
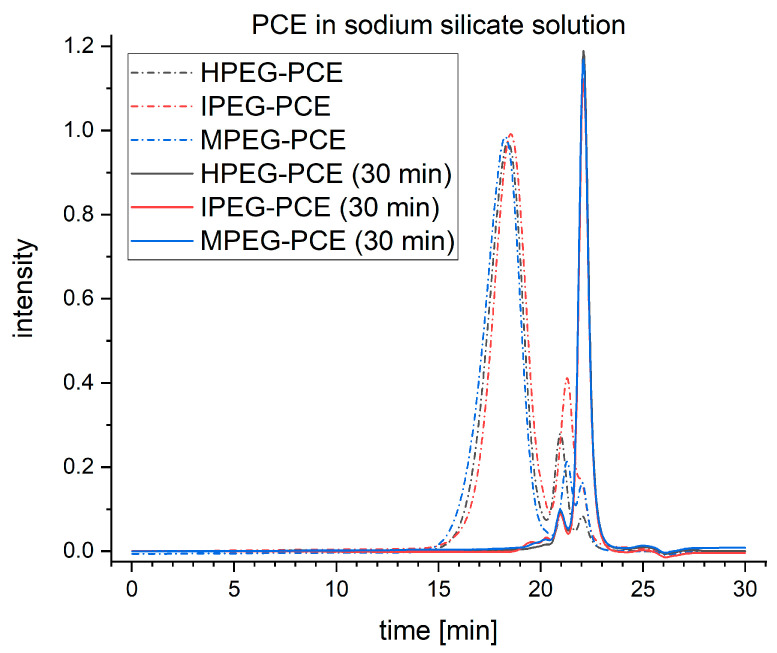
Elugrams of reference PCEs and PCEs exposed to sodium silicate solution for 30 min.

In comparison to the alkali hydroxide solutions, the degradation process of the PCEs was found to be much stronger in the alkali silicate solutions. This corresponds to the results reported by Palacios et al., who found that commercial superplasticizers were ineffective, especially in sodium silicate solutions [36]. Figure 8 shows the exemplary elugrams for the samples exposed to a sodium silicate solution for 30 min.

**Figure 9:**
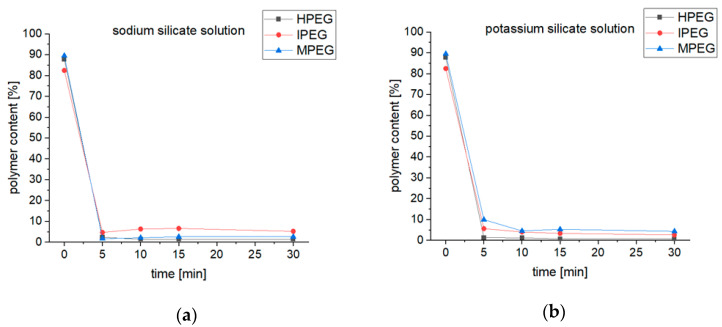
Polymer content of PCE samples exposed to sodium silicate solution (**a**) and potassium silicate solution (**b**) over time.

The disappearance of the first peak of all the samples after exposure to the alkaline medium indicates that most of the PCEs were degraded to PEG sidechains and the respective polyacrylic backbones. The second peak is significantly smaller after the exposure, which could be assigned to the degradation of the macromonomers to PEG and the respective acrylic acid derivatives. These apparently have a similar molecular mass to the PEG side chains. As expected, the third peak grows much larger in size due to the degradation process of the polymers and macromonomers. The calculated polymer content of the samples exposed to the alkaline medium is plotted in Figure 9, and a drastic decrease in the polymer content to <10% is shown, while the monomer content increases to >90% after five minutes of exposure. With a longer exposure, no significant change in the polymer content can be seen for most of the samples. However, the MPEG-PCE samples’ polymer content decreases from ≈10% to ≈5% going from 5 to 10 min of exposure to the alkaline medium.

**Figure 10:**
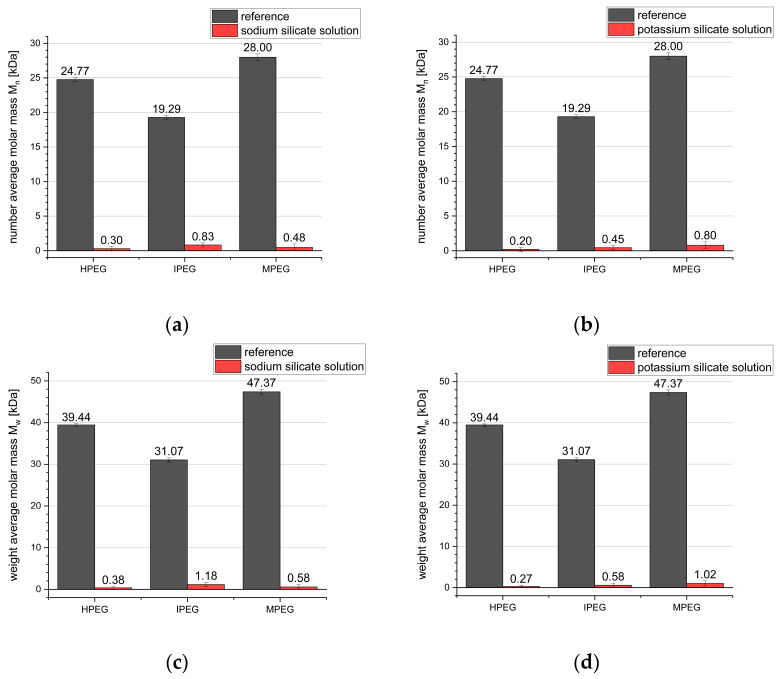
Number average molar mass (**a**) sodium silicate solution, (**b**) potassium silicate solution and weight average molar mass (**c**) sodium silicate solution, (**d**) potassium silicate solution of the PCE reference and samples exposed to alkali silicate solutions for 30 min.

Figure 10 shows a comparison of the M_n_ and M_w_ for the samples exposed to the alkali silicate solution for 30 min, with concentrations of 7.8 M for the sodium silicate solution and 6.6 M for the potassium silicate solution. A strong decrease in both values to >2 kDa can be seen, regardless of the type of alkali silicate solution. Although these concentrations of alkali hydroxide are comparable to the investigated alkali hydroxide solution concentrations, a much stronger degree of degradation was seen. This is most likely linked to the presence of silicate ions and will be further investigated.

## 4. Conclusions

The study involved systematic stability investigations of ester and ether-type polycarboxylate superplasticizers (MPEG, HPEG and IPEG PCEs) in high-alkaline activators for low-calcium geopolymers. In particular, the type (NaOH, KOH sodium and potassium silicate solutions), alkalinity and exposure time in the alkaline activator play an important role in the hydrolyzation of the polyethylene glycol side chains of PCEs. By size exclusion chromatography, it could be shown that all the investigated PCEs will be degraded independent of the alkalinity of the sodium hydroxide and potassium hydroxide solutions. In particular, for a 1 M sodium hydroxide and potassium hydroxide solution, the degradation process is dependent on the time of exposure. At higher alkalinity (4 M and 8 M), degradation occurs in the first 5 min of exposure and leads to a reduction of the molecular weight of approx. 45–55%. When exposed to alkali silicate solutions, the degree of degradation of the PCEs was found to be much higher, so the polymer content was reduced to >10% within the first five minutes. The M_n_ and M_w_ were reduced to <2 kDa. The reason for the low efficiency of the PCE superplasticizers is the hydrolyzation of the ether and ester-bonded side chains. By rheological investigations, it could be shown that all the investigated PCEs generated an increase in the viscosity and yield strength of the geopolymer paste. This is due to the free polyethylene glycol side chains, which are responsible for the thickening of the geopolymer paste. Therefore, it will be necessary to develop efficient and environmentally friendly superplasticizers that exhibit high stability in alkaline activators of future binder systems.

## Figures and Tables

**Table 1:** Number average and weight average molar mass, PDI and polymer content of investigated PCE samples.

	HPEG-PCE	IPEG-PCE	MPEG-PCE
Mn [kDa]	25.0 ± 0.3	19.4 ± 0.5	28.1 ± 0.6
Mw [kDa]	39.6 ± 0.3	31.6 ± 0.3	46.6 ± 0.5
PDI	1.59 ± 0.0	1.63 ± 0.0	1.66 ± 0.0
polymer content [%]	87.7	82.4	89.5

**Table 2:** Number average molar mass and weight average molar mass of the PCE references and samples exposed to alkali hydroxide solutions for 30 min.

	HPEG-PCE Mn/Mw [kDa]	IPEG-PCE Mn/Mw [kDa]	MPEG-PCE Mn/Mw [kDa]
**Reference**	25.0/39.6	19.4/31.6	28.1/46.6
1 M NaOH	21.4/37.5	17.3/27.9	20.0/33.0
4 M NaOH	19.2/36.7	14.9/23.9	17.6/31.5
8 M NaOH	16.7/31.2	13.0/23.0	14.0/22.8
1 M KOH	22.2/38.6	17.8/25.3	26.8/43.9
4 M KOH	20.2/31.8	15.1/24.2	24.7/40.1
8 M KOH	17.9/28.8	13.6/22.4	16.4/27.6

## Data Availability

The data presented in this study are available from the corresponding authors upon reasonable request.
